# Supplementary material for: Investigation of the Membrane Fluidity Regulation of Fatty Acid Intracellular Distribution by Fluorescence Lifetime Imaging of Novel Polarity Sensitive Fluorescent Derivatives
Source: Int J Mol Sci. 2021 Mar 18;22(6):3106. doi: 10.3390/ijms22063106 (PMC8002861; doi:10.3390/ijms22063106)
Supplement: Supplementary file 1 [file ijms-22-03106-s001.zip › FAderivatives_ijms-Supplementary.docx]

Supplementary Materials

Investigation of the membrane fluidity regulation of fatty acid intracellular distribution by fluorescence lifetime imaging of novel polarity sensitive fluorescent derivatives

Giada Bianchetti ^1,2^, Salome Azoulay-Ginsburg ^3^, Nimrod Yosef Keshet-Levy ^3,4^, Aviv Malka ^3^, Sofia Zilber ^4^, Edward E. Korshin ^3^, Shlomo Sasson ^5^, Marco De Spirito ^1,2^, Arie Gruzman ^3,*^, and Giuseppe Maulucci ^1,2,^*^,+^

^1^	Neuroscience Department, Biophysics Section, Università Cattolica del Sacro Cuore, 00168, Rome, IT; [giada.bianchetti@unicatt.it](mailto:giada.bianchetti@unicatt.it); [marco.despirito@unicatt.it](mailto:marco.despirito@unicatt.it)

**Citation:** Bianchetti, G.; Azoulay-Ginsburg, S.; Keshet-Levy, N.Y. et al. Investigation of the membrane fluidity regulation of fatty acid intracellular distribution by fluorescence lifetime imaging of polarity sensitive fluorescent analogs. *Int. J. Mol. Sci.* **2021**, *22*, x. https://doi.org/10.3390/xxxxx

Academic Editor: Firstname Lastname

Received: date

Accepted: date

Published: date

**Publisher’s Note:** MDPI stays neutral with regard to jurisdictional claims in published maps and institutional affiliations.

**Copyright:** © 2021 by the authors. Submitted for possible open access publication under the terms and conditions of the Creative Commons Attribution (CC BY) license (http://creativecommons.org/licenses/by/4.0/).

^2^	Fondazione Policlinico Universitario “A. Gemelli” IRCSS, 00168, Rome, IT

^3^	Department of Chemistry, Bar-Ilan University, 59290002, Ramat Gan, IL; SAG: [salomeazoulay16@gmail.com](mailto:salomeazoulay16@gmail.com); NYKL: [labpath@szmc.org.il](mailto:labpath@szmc.org.il); AM: [aviv1m@walla.co.il](mailto:aviv1m@walla.co.il); EK: [edward.korshin@gmail.com](mailto:edward.korshin@gmail.com); AG: [gruzmaa@biu.ac.il](mailto:gruzmaa@biu.ac.il)

^4^	Department of Pathology, Shaare Zedek Medical Center, 9103102, Jerusalem, IL; [sofiazilber@szmc.org.il](mailto:sofiazilber@szmc.org.il)

^5^	Institute for Drug Research, Faculty of Medicine, The Hebrew University, 911210 Jerusalem, IL; [shlomo.sasson@mail.huji.ac.il](mailto:shlomo.sasson@mail.huji.ac.il)

* Senior Authors

^+^ Correspondence: GM: [giuseppe.maulucci@unicatt.it](mailto:giuseppe.maulucci@unicatt.it); Tel.: +39 06 3015 4265

***Characterization of synthesized compounds - NMR Spectra Data***

**Figure S1. ^1^H NMR (300 MHz, CDCl_3_) – (1)**

**Figure S2. ^13^C NMR (75 MHz, CDCl_3_) – (1)**

**Figure S3. DEPT NMR (75 MHz, CDCl_3_) – (1)**

**Figure S4. ^1^H NMR (400 MHz, CDCl_3_) – (2)**

**Figure S5. ^13^C NMR (100 MHz, CDCl_3_) – (2)**

**Figure S6. DEPT NMR (75 MHz, CDCl_3_) – (2)**

Acetone

**Figure S7. ^1^H NMR (300 MHz, acetone-d_6_) – (3)**

**Figure S8.** **^1^H NMR (300 MHz, CDCl_3_ – (4)**

**Figure S9. ^13^C NMR (75 MHz, CDCl_3_) – (4)**

**Figure S10. DEPT NMR (75 MHz, CDCl_3_) – (4)**

**Figure S11. ^1^H NMR (400 MHz, acetone-d_6_) – (SG14:0)**

**Figure S12. ^13^C NMR (100 MHz, acetone-d_6_) – (SG14:0)**

**Figure S13. DEPT NMR (100 MHz, acetone-d_6_) – (SG14:0)**

**Figure S14. COSY (400 MHz, acetone-d_6_) – (SG14:0)**

**Figure S15. HMQC (100 MHz, acetone-d_6_) - (SG14:0)**

**Figure S16. HMBC (100 MHz, acetone-d_6_) - (SG14:0)**

**Figure S17. ^1^H NMR (600 MHz, acetone-d_6_) – (SG16:0)**

**Figure S18. ^13^C NMR (150 MHz, acetone-d_6_) – (SG16:0)**

**Figure S19. DEPT NMR (100 MHz, acetone- d_6_) – (SG16:0)**

**Figure S20. COSY (400 MHz, CDCl_3_) - (SG16:0)**

**Figure S21. HMQC (100 MHz, CDCl_3_) - (SG16:0)**

**Figure S22. HMBC (100 MHz, CDCl_3_) - (SG16:0)**

**Figure S23. ^1^H NMR (400 MHz, CDCl_3_) – (SG18:0)**

**Figure S24. ^13^C NMR (100 MHz, CDCl_3_) – (SG18:0)**

**Figure S25. DEPT NMR (100 MHz, CDCl_3_) – (SG18:0)**

**Figure S26. COSY (400 MHz, CDCl_3_) – (SG18:0)**

**Figure S27. HMQC (100 MHz, CDCl_3_) - (SG18:0)**

**Figure S28. HMBC (100 MHz, CDCl_3_) - (SG18:0)**

***Characterization of synthesized compounds – High resolution Mass Spectroscopy (HRMS) data***

**Figure S29. High resolution Mass Spectroscopy (HRMS) data - (1)**

**Figure S30. High resolution Mass Spectroscopy (HRMS) data - (2)**

**Figure S31. High resolution Mass Spectroscopy (HRMS) data - (4)**

**Figure S32. High resolution Mass Spectroscopy (HRMS) data - (SG14:0)**

**Figure S33. High resolution Mass Spectroscopy (HRMS) data - (SG16:0)**

**Figure S34. High resolution Mass Spectroscopy (HRMS) data - (SG18:0)**
